# Commentary: Human Liver Flukes

**DOI:** 10.3389/fpubh.2018.00122

**Published:** 2018-04-30

**Authors:** Monica Catarina Botelho, Francisco Almeida, Joachim Richter, Antonio Sarmento

**Affiliations:** ^1^Instituto Nacional de Saude Dr. Ricardo Jorge, Porto, Portugal; ^2^i3S, Instituto de Investigação e Inovação em Saúde, Porto, Portugal; ^3^Centro Hospitalar São João, Porto, Portugal; ^4^Institut für Tropenmedizin und Internationale Gesundheit Charité, Universitätsmedizin Berlin, Berlin, Germany

**Keywords:** fascioliasis, *Fasciola hepatica*, Porto, Portugal, infection

We read with interest the paper by Harrington et al. (1) on “human liver flukes,” recently published in the Lancet Gastroenterology and Hepatology. They report that the prevalence of *Fasciola hepatica* in the inner Porto area of Portugal is 3.2% (1). These authors also state that this is a notably high prevalence (1).

Fascioliasis, as a neglected tropical disease, commonly affects poor people from developing countries and occurs sporadically in Europe (2). In 1996, Dias et al. (3) reported a case of chronic fascioliasis in a patient with a 6-year history of intermittent biliary colic after having ingested uncooked wild watercress (3). Our group has performed a search in the database of Centro Hospitalar de S. João (CHSJ), from 1997 to 2017. CHSJ is the biggest hospital in the North of Portugal. This hospital is located in the city of Porto and serves a population of 1,700,000 inhabitants distributed by an approximate area of 2,040 km^2^. The results we have obtained diverge from the figures reported by Harrington et al. (1): during the last 20 years only four cases of fascioliasis have been recorded: one case in 1998, one case in 2003, and two cases in 2014.

The high prevalence of fascioliasis reported by Harrington and colleagues (1) in comparison with the only sporadic occurrence of fascioliasis in our actual database search, suggests that the frequency of fascioliasis has largely decreased at least in Northern Portugal. This assumption is corroborated by the fact that Harrington et al. (1) based their conclusions on a review of Mas-Coma et al. from 1999 (4). Considering that this reference is older than 25 years and that the hygienic and living standards of the population of the North of Portugal evolved considerably in these years, as well as in keeping with Harrington et al. (1) who have, themselves, noted that the estimates of prevalence of human liver fluke infections are, in most cases, decades old and probably inaccurate, our up-to-date figures appear to reflect more reliably the actual situation.

Although fascioliasis remains a public health problem in many areas of the world, we now provide actual information on the knowledge of the true distribution of *F. haepatica* in the inner Porto area of Portugal.

## Author Contributions

MB: designed and wrote. FA: performed the analysis. JR and AS: critically read the paper.

## Conflict of Interest Statement

The authors declare that the research was conducted in the absence of any commercial or financial relationships that could be construed as a potential conflict of interest.
